# Effect of Inflammatory Signaling on Human Articular Chondrocyte Hypertrophy: Potential Involvement of Tissue Repair Macrophages

**DOI:** 10.1177/19476035211021907

**Published:** 2021-06-24

**Authors:** Mauricio N. Ferrao Blanco, Yvonne M. Bastiaansen-Jenniskens, Mark G. Chambers, Andrew A. Pitsillides, Roberto Narcisi, Gerjo J.V.M. van Osch

**Affiliations:** 1 Department of Orthopaedics and Sports Medicine, Erasmus MC, University Medical Center Rotterdam, Rotterdam, The Netherlands; 2 Lilly Research Laboratories, Eli Lilly Pharmaceuticals, Indianapolis, IN, USA; 3 Comparative Biomedical Sciences, Royal Veterinary College, London, UK; 4 Department of Otorhinolaryngology, Erasmus MC, University Medical Center Rotterdam, Rotterdam, The Netherlands; 5 Department of Biomechanical Engineering, TU Delft, Delft, The Netherlands

**Keywords:** osteoarthritis, articular chondrocytes, hypertrophy, inflammation, macrophages

## Abstract

**Objective:**

In osteoarthritis, chondrocytes tend to acquire a hypertrophic phenotype, which contributes to the modification of the extracellular matrix, resulting in permanent cartilage changes. In mouse chondrocytes, pro-inflammatory macrophages and pro-inflammatory cytokines have been shown to stimulate hypertrophy via the activation of the nuclear factor kappa B (NF-κB) pathway. Whether or not this also occurs in human chondrocytes remains unclear. We therefore aimed to investigate whether hypertrophy-like responses in human cartilage are driven mainly by intrinsic inflammatory signaling or shaped by specific macrophage populations.

**Design:**

Human articular chondrocytes were cultured with pro-inflammatory cytokines or medium conditioned by defined macrophage subsets. Furthermore, the effect of inhibition of NF-κB-dependent gene expression was evaluated using the NF-κB inhibitor SC-514. Hypertrophy was assessed by measuring the transcription level of alkaline phosphatase (*ALPL*), type X collagen (*COL10A1*), Indian hedgehog (*IHH*), and runt-related transcription factor 2 (*RUNX2*).

**Results:**

The expression of hypertrophic genes was not promoted in human chondrocytes by pro-inflammatory cytokines neither pro-inflammatory M(IFNγ + TNFα) macrophages. Inhibition of the NF-κB-dependent gene expression did not affect human articular chondrocyte hypertrophy. However, tissue repair M(IL4) macrophages induced hypertrophy by promoting the expression of *COL10A1*, *RUNX2*, and *IHH*.

**Conclusion:**

Intrinsic inflammatory signaling activation is not involved in the hypertrophic shift observed in human articular chondrocytes cultured *in vitro*. However, tissue repair macrophages may contribute to the onset of this detrimental phenotype in human osteoarthritic cartilage, given the effect observed in our experimental models.

## Introduction

Osteoarthritis (OA) is characterized by progressive loss of articular cartilage, formation of osteophytes, degeneration of the ligaments, and inflammation of the synovium. Even though significant progress has been made in OA research in recent years, advances are still needed to understand the molecular mechanisms of OA in order to develop therapeutic strategies. Articular chondrocytes are responsible for maintaining the balance between catabolic and anabolic processes in the cartilage. In OA, this homeostatic state is lost and chondrocytes acquire an altered phenotype, promoting the degradation of the cartilage and vascularization.^1-3^ These hypertrophic-like chondrocytes are characterized by the expression of alkaline phosphatase (*ALPL*), type X collagen (*COL10A1*), Indian hedgehog (*IHH*), matrix metalloproteinase 13 (*MMP13*), and runt-related transcription factor 2 (*RUNX2*).^4,5^

Increased attention has been paid to the inflammatory process in OA, not only in the symptomatology but also in the pathophysiology of disease initiation and progression.^6,7^ Interestingly, inflammatory signaling activation can direct mouse chondrocytes toward hypertrophic differentiation through the nuclear factor kappa B (NF-κB) pathway, which has a major role in the progression of OA in mice models.^8,9^ Macrophages play a prominent role in the progression of OA and are the dominant leukocyte population in inflamed osteoarthritic synovium.^10-12^ Macrophages are plastic cells that can acquire a pro- or anti-inflammatory phenotype, depending on environmental cues.^13^ Pro-inflammatory macrophages induced the upregulation of catabolic enzymes in human articular chondrocytes^14^ and have been shown to promote hypertrophy in mouse chondrocytes.^15^ Here we sought to understand whether chondrocyte hypertrophic-like responses in human cartilage are driven mainly by intrinsic inflammatory signaling, as in mouse, or shaped by specific macrophage populations.

## Methods

### Cartilage Explant and Chondrocyte Isolation

Human articular cartilage was obtained with implicit consent as waste material from patients undergoing total knee replacement surgery (9 females, 5 males, 67 ± 11 years). This protocol was approved by the Medical Ethical Committee of the Erasmus MC, University Medical Center, Rotterdam, protocol number MEC-2004-322. Full thickness cartilage explants (ø = 5 mm) were harvested from macroscopically intact areas and washed twice with 0.9% NaCl (Sigma Aldrich, St. Louis, MO, USA). To isolate chondrocytes, cartilage chips were subjected to protease (2 mg/mL, Sigma Aldrich) for 2 hours followed by overnight digestion with 1.5 mg/mL collagenase B (Roche Diagnostics, Basel, Switzerland) in Dulbecco’s modified Eagle’s medium (DMEM) high glucose supplemented with 10% fetal bovine serum. Single cell suspension was obtained by filtrating the cellular solution by a 100 µm filter. The isolated chondrocytes were expanded in monolayer at a seeding density of 7,500 cells/cm^2^ in DMEM high glucose supplemented with 10% fetal bovine serum, 50 μg/mL gentamicin, and 1.5 μg/mL fungizone (Gibco, Grand Island, NY, USA). Approximately 80% confluency cells were trypsinized and reseeded at 7,500 cells/cm^2^. Cells were used for experiments after 3 passages.

### Preparation of Macrophage Conditioned Medium

Monocytes were isolated from 2 buffy coats (males, 54 and 58 years, Sanquin, Amsterdam, the Netherlands) using Ficoll (GE Healthcare, Little Chalfont, UK) density gradient separation and cluster of differentiation (CD)14 magnetic-activated cell sorting microbeads (MACS; Miltenyi, Bergisch Gladbasch, Germany). To prepare macrophage conditioned medium (MCM), monocytes were seeded in culture flasks at 500,000 monocytes/cm^2^ and cultured in X-VIVO TM-15 (Lonza, Verviers, Belgium) containing 20% heat-inactivated fetal calf serum (FCS; Lonza), 50 μg/mL gentamicin (Gibco), and 1.5 μg/mL fungizone (Gibco) at 37 °C and 5% CO_2_. Monocytes were stimulated with 10 ng/mL interferon-γ (IFNγ; PeproTech, Rocky Hill, NJ, USA) and 10 ng/mL tumor necrosis factor-α (TNFα, PeproTech) to obtain pro-inflammatory M(IFNγ + TNFα) macrophages. Tissue repair M(IL-4) macrophages were obtained after stimulation with 10 ng/mL interleukin-4 (IL-4; PeproTech) and anti-inflammatory M(IL-10) macrophages were acquired by stimulation with 10 ng/mL IL-10 (PeproTech). After 72 hours, medium and stimuli were renewed and after 24 hours the medium was removed, the macrophages were washed twice with 0.9% NaCl and subsequently cultured in serum-free DMEM low glucose supplemented with 1% insulin-transferrin-selenium (ITS premix, BD Biosciences, San Jose, CA, United States), 50 μg/mL gentamicin, 1.5 µg/mL fungizone, and 25 µg/mL l-ascorbic acid 2-phosphate (Sigma Aldrich) to obtain MCM. After 24 hours, the MCM was harvested, centrifuged at 200 × *g* and stored at −80 °C until use. The media conditioned by M(IFNγ + TNFα), M(IL4), and M(IL10) macrophages were confirmed to contain a higher concentration of IL-6, CCL18, and sCD163, respectively (Supplementary Figure 1), in accordance with our previous work.^16-19^ Nonconditioned DMEM low glucose supplemented with 1% ITS premix, 50 µg/mL gentamicin, 1.5 µg/mL fungizone, and 25 µg/mL l-ascorbic acid 2-phosphate was also incubated, centrifuged, and frozen to serve as control medium. Cells were harvested for DNA quantification with a modified CyQUANT assay (Invitrogen, Carlsbad, CA, USA). All MCM used for culture and analysis were frozen and thawed once. For further experiments 50% of MCM or 50% nonconditioned medium was mixed with 50% fresh medium to replenish potentially depleted nutrients.

### Exposure of Chondrocytes and Cartilage Explants to Inflammatory Cytokines and Macrophage Conditioned Medium

In order to select an inflammatory stimulus, passage three chondrocytes cultured in a 6-well plate (BD Falcon, Bedford, MA, USA) at a seeding density of 20,000 cells/cm^2^ were exposed to pro-inflammatory cytokines (IL-1β, TNF-α, or IFN-γ) at 1 ng/mL and, alternatively, to a combination of the 3 cytokines, each at 0.1 ng/mL. The combination of pro-inflammatory cytokines was selected based on a pilot experiment where nitric oxide (NO) was measured in the media as measurement of induction of inflammation (Supplementary Figure 2). Pro-inflammatory cytokines alone at 1 ng/mL did not significantly increase NO in the medium, which might be due to the basal NO production in OA chondrocytes.

To accommodate the likelihood that the acquisition of more fibroblast-like phenotype by these passage 3 chondrocytes may modify these responses, we examined behavior following redifferentiation. Briefly, redifferentiation of articular chondrocytes was performed using the well-established 3-dimensional alginate bead culture model,^20^ and confirmed by *COL2A1* expression. Moreover, our data show that OA chondrocytes express the hypertrophic markers *COL10A1* and *RUNX2*, being a suitable model to study chondrocyte hypertrophy. For preparation of alginate beads, chondrocytes after 3 passages in monolayer were resuspended in 1.2% (w/v) low-viscosity alginate (Kelton LV alginate, Kelko Co, San Diego, CA, USA) in 0.9% NaCl (Sigma Aldrich) at a concentration of 4 × 10^6^ cells/mL. Beads were made by dripping the cell-alginate suspension in 105 mM CaCl_2_ (Sigma Aldrich) through a 22-gauge needle. Beads were washed with 0.9% NaCl and DMEM low glucose. Beads with a size that deviated from the average after a visual inspection were not included in the experiment. Redifferentiation of chondrocytes was performed in a 24-well plate (BD Falcon) for 2 weeks in 100 μL/bead DMEM low glucose supplemented with 1% ITS fetal (Biosciences), 10 ng/mL transforming growth factor beta 1 (TGF-β1, recombinant human, R&D systems) 25 μg/mL l-ascorbic acid 2-phosphate (Sigma Aldrich), 50 μg/mL gentamicin, and 1.5 μg/mL fungizone (both Gibco). After 2 weeks, TGF-β1 was no longer added to the medium and cells were either cultured with 10 µM of the NFkB inhibitor SC-514 (Cayman Chemicals, Ann Arbor, MI, USA) for 24 hours or with the combination of pro-inflammatory cytokines TNFα, IFNγ, IL-1β at 0.1 ng/mL for 1 week, refreshing the medium twice.

Cartilage explants were cultured in DMEM low glucose supplemented with 1% ITS premix, 50 μg/mL gentamicin, and 1.5 μg/mL fungizone and either a combination of pro-inflammatory cytokines TNFα, IFNγ, IL-1β at 0.1 ng/mL each or medium conditioned by macrophages during 1 week, refreshing the medium twice.

### Nitric Oxide Assay

NO production was measured in the medium of OA chondrocytes by determining the content of nitrite using the Griess reagent (Sigma Aldrich). The reaction was monitored at 540 nm using a spectrophotometer (VersaMax; Molecular Devices, Sunnyvale, CA, USA). Sodium nitrite (NaNO_2_; Chemlab, Zedelgem, Belgium) was used as standard.

### Gene Expression

Alginate beads were dissolved using citrate buffer, centrifuged at 200 × *g* and the pellet was resuspended in RLT (Qiagen, Hilden, Germany) buffer containing 1% β-mercaptoethanol for RNA isolation. RNA was isolated from the cartilage explants by snap freezing in liquid nitrogen followed by pulverization using a Mikro-Dismembrator (B. Braun Biotech International GmbH, Melsungen, Germany) at 2800 rpm. The tissue was homogenized with 18 μL/mg sample RNA-Bee TM (Tel-Test Inc., Friendswood, TX, USA) and 20% chloroform. mRNA isolation was performed according to manufacturer’s protocol utilizing the RNeasy column system (Qiagen, Hilden, Germany). The RNA concentration was determined using a NanoDrop spectrophotometer (Isogen Life Science, Utrecht, the Netherlands). 0.5 μg RNA was used for cDNA synthesis following the protocol of the manufacturer of the RevertAid First Strand cDNA kit (Thermo Fisher Scientific, Waltham, MA, United States). Quantitative polymerase chain reaction (qPCR) was performed on a Bio-Rad CFX96 Real-Time PCR Detection System (Bio-Rad) to assess gene expression, alkaline phosphatase (*ALPL*, Fw: GACCCTTGACCCCCACAAT; Rev: CTCGTACTGCATGTCCCCT; Probe: TGGACTACCTATTGGGTCTCTTCGAGCCA), Collagen type 2 (*COL2A1*; Fw: GGCAATAGCAGGTTCACGTAC; Rev: CGATAACAGTCTTGCCCCACTT; Probe: CCGGTATGTTTCGTGCAGCCATCCT), Collagen type 10 (*COL10A1*; Fw: CAAGGCACCATCTCCAGGAA; Rev: AAAGGGTATTTGTGGCAGCATATT; Probe: TCCAGCACGCAGAATCCATCTGA), matrix metalloproteinase-13 (*MMP13*; Fw: AAGGAGCATGGCGACTTCT; Rev: TGGCCCAGGAGGAAAAGC; Probe: CCCTCTGGCCTGCGGCTCA), Runt-related transcription factor 2 (*RUNX2*; Fw: ACGTCCCCGTCCATCCA; Rev: TGGCAGTGTCATCATCTGAAATG; Probe: ACTGGGCTTCCTGCCATCACCGA), Tumor Necrosis Factor-a (*TNFA*; Fw: GCCGCATCGCCGTCTCCTAC; Rev: AGCGCTGAGTCGGTCACCCT). Indian hedgehog (*IHH*) primer was purchased as assays-on-demand from BioRad. Glyceraldehyde-3-phosphate dehydrogenase (*GAPDH*; Fw: ATGGGGAAGGTGAAGGTCG; Rev: TAAAAGCAGCCCTGGTGACC; Probe: CGCCCAATACGACCAAATCCGTTGAC) was found stable and therefore used as reference gene. Data were analyzed by the ΔΔCt method and normalized to the expression of *GAPDH* of each condition and compared to the corresponding gene expression in the control groups. Articular cartilage explants were divided in hypertrophic and nonhypertrophic donors based on the expression of the hypertrophic markers *COL10A1*, *RUNX2*, and *IHH* in the basal – control condition, by using a cycle cutoff of 36. Donors with Cq of 36 or higher were classified as nonhypertrophic.

### Statistics

Each experiment included at least 3 technical replicates and was repeated with cells/explants derived from at least 3 OA donors. Statistical evaluation was performed using IBM SPSS 22.0. The normal distribution of the data was confirmed using the Kolmogorov-Smirnov test. The linear mixed model was applied using the different conditions as fixed parameters and the donors as random factors.

## Results

### Pro-inflammatory Cytokines Did Not Promote Hypertrophy in Human Chondrocytes *In Vitro*

**Figure 1.**
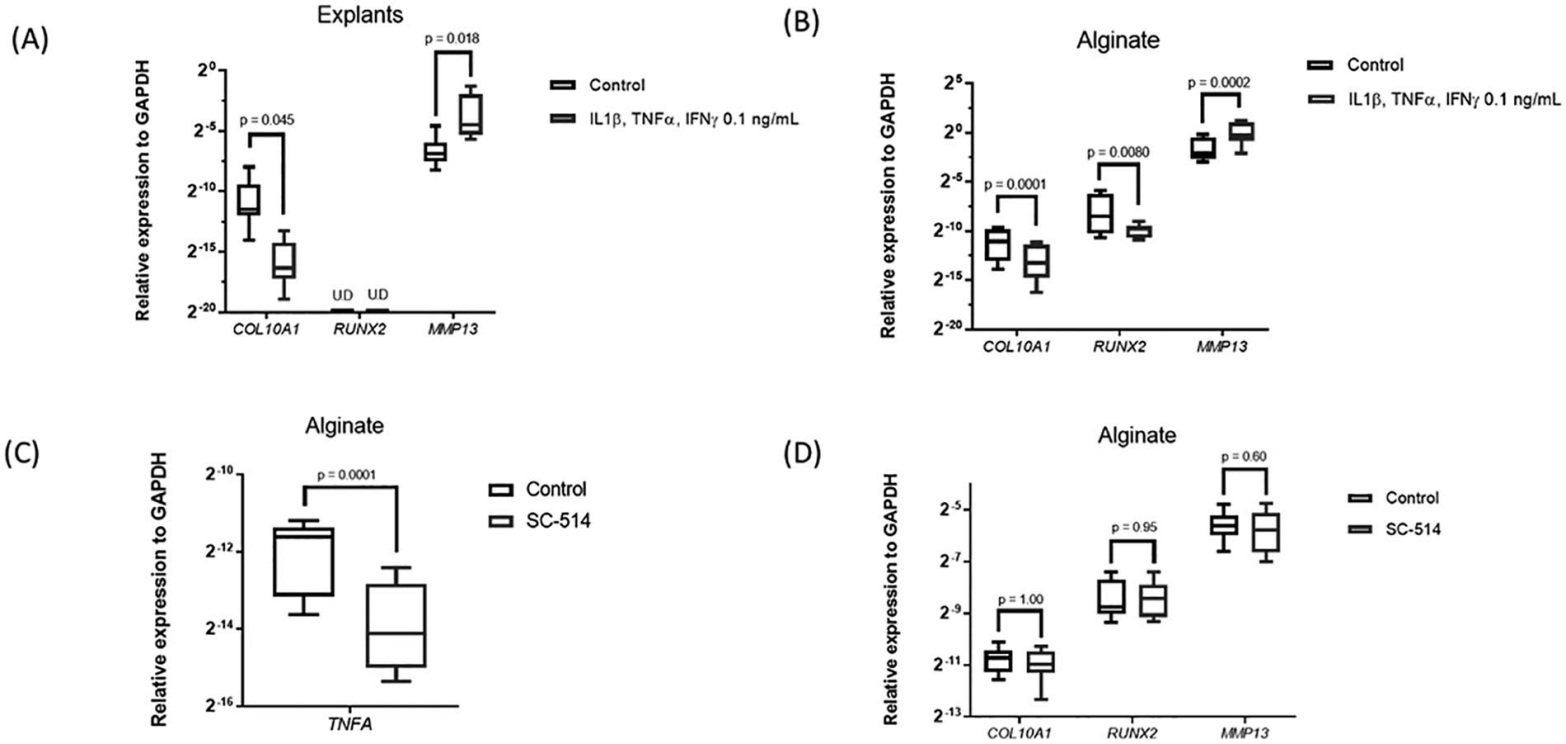
Effect of pro-inflammatory signal activation in chondrocytes hypertrophy. (**A**) Osteoarthritic (OA) human cartilage explants and (**B**) chondrocytes encapsulated in alginate stimulated with the combination of the inflammatory cytokines at 0.1 ng/mL for 1 week (*n* = 3 donors, 3 samples per donor). (**C** and **D**) OA human chondrocytes encapsulated in alginate cultured with the NFκB inhibitor, SC-514 at 10 µM for 24 hours (*n* = 3 donors, 3 samples per donor). UD = undetectable. Data are shown as minimum to maximum.

To study the effect of pro-inflammatory signaling activation on chondrocyte hypertrophy, we used a combination of inflammatory cytokines that are secreted by macrophages, IL1β, TNFα, and IFNγ and used 2 different models, human articular cartilage explants and human articular chondrocytes in alginate. On inflammatory stimulation, the expression of the catabolic enzyme *MMP13* was increased in cartilage explants and chondrocytes in alginate (**Fig. 1A** and **B**). Interestingly, the expression of the hypertrophic marker *COL10A1* was significantly decreased in both models. *RUNX2* was downregulated in alginate on cytokine addition and not detectable in explants. *IHH* and *ALPL* were not detectable in either of the models. To evaluate whether endogenous inflammation present in osteoarthritic chondrocytes influenced hypertrophy, the NF-κB inhibitor SC-514 was added to alginate cultures. SC-514 significantly decreased mRNA expression of the NFκB-dependent gene *TNFA*, confirming its efficacy (**Fig. 1C**). However, NFκB inhibition did not modify *COL10A1*, *RUNX2*, or *MMP13* expression, indicating that hypertrophy is not affected in osteoarthritic chondrocytes when the main inflammatory pathway is inhibited (**Fig. 1D**). The expression of *MMP13* is related to hypertrophy and to inflammatory signaling in chondrocytes. The absence of an effect of SC-514 on *MMP13* expression thus suggests that *MMP13* in these osteoarthritic chondrocytes is regulated by other transcription factors such as β-catenin.^21,22^ Summarizing, these data indicate that pro-inflammatory signaling did not stimulate a hypertrophic phenotype in human articular chondrocytes *in vitro*.

### Tissue Repair M(IL4) Macrophages Are Associated with the Onset of Human Chondrocyte Hypertrophy *In Vitro*

**Figure 2.**
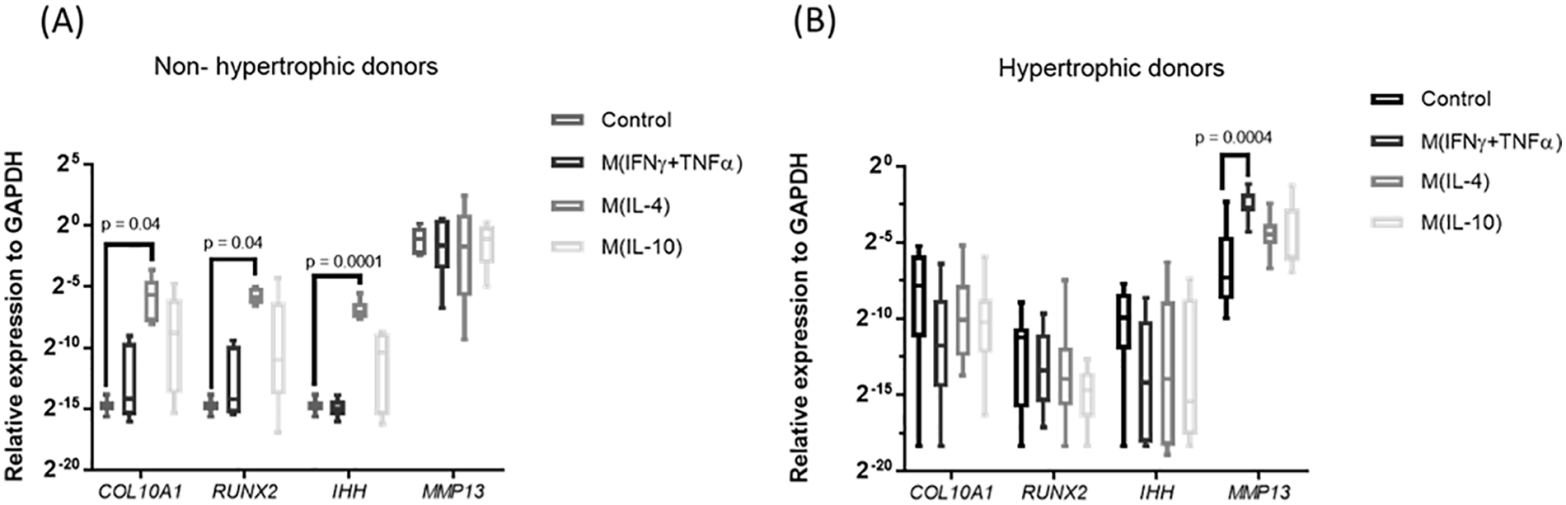
Effect of macrophage secretome on chondrocytes hypertrophy. (**A**) Nonhypertrophic osteoarthritic (OA) human cartilage explants stimulated with macrophage condition medium (*n* = 2 donors, 3 samples per donor). (**B**) Hypertrophic OA human cartilage explants stimulated with macrophage condition medium (*n* = 3 donors, 3 samples per donor) Data are shown as minimum to maximum. Articular cartilage explants were divided in hypertrophic and non-hypertrophic donors based on the expression of the hypertrophic markers *COL10A1*, *RUNX2*, and *IHH* in the basal – control condition, by using a cycle cutoff of 36. Donors with Cq of 36 or higher were classified as nonhypertrophic.

To better mimic the complex combination of inflammatory factors in the joint, the medium conditioned by different macrophage phenotypes was evaluated for its capacity to modulate chondrocyte hypertrophy. Medium conditioned by pro-inflammatory M(IFNγ + TNFα) or anti-inflammatory M(IL10) macrophages had no effect on expression of *COL10A1*, *RUNX2*, *IHH*, or *ALPL* in cartilage explants (**Fig. 2**). Medium conditioned by tissue repair M(IL4) macrophages, however, significantly upregulated the expression of *COL10A1, RUNX2*, and *IHH* in explants of 2 of the 5 experiments. These 2 were nonhypertrophic basally, but the remaining 3 exhibited basal hypertrophy. These data suggest that pro-inflammatory factors do not induce hypertrophic differentiation of human articular chondrocytes, but factors secreted by tissue repair macrophages can induce hypertrophy.

## Discussion

Inflammation-induced hypertrophy is a process that has been suggested to play role in the progression of osteoarthritis, but current studies mainly focused on mouse chondrocytes.^8,9^ The findings of our study demonstrate that pro-inflammatory cytokines do not mediate the hypertrophic differentiation of human articular chondrocytes *in vitro*. Inflammatory processes in OA are mainly driven by macrophages, which generate a broad spectrum of cytokines and immune factors. Previous studies in mouse have shown that pro-inflammatory macrophages induced hypertrophy.^15^ However, here we show that pro-inflammatory macrophages do not increase hypertrophy of human chondrocytes. In addition, although previous studies in mice chondrocytes have shown that the NF-κB pathway is responsible for inflammation-induced hypertrophy,^23^ here we showed that inhibition of NF-κB did not reduce hypertrophy in human osteoarthritic chondrocytes. These results indicate that, differently from murine chondrocytes, pro-inflammatory signaling cues do not unavoidably induce hypertrophy in human chondrocytes that have been isolated from osteoarthritic knee joints and subsequently maintained *in vitro*.

Macrophages can acquire different phenotypes depending on the environmental stimuli, hence secreting cytokines that lead to various responses in the tissue. Our data suggest that tissue repair macrophages can induce a phenotypic shift in articular chondrocytes toward a hypertrophy state. A limitation of our study is that the number of nonhypertrophic OA donors was low, probably due to the late stage of disease in the majority of OA donors that undergo total knee replacement. These donors had a higher basal *MMP13* expression compared with the hypertrophic donors, which might suggest that they had a higher basal inflammatory state. Even though the numbers are low, this demonstrates a proof of principle that macrophages with tissue repair phenotype have the capacity to induce hypertrophy. Interestingly, this macrophage subset secretes the cytokine TGFβ, which has been associated to hypertrophy in aging cartilage as well as in articular chondrocytes in culture.^24,25^ Current literature in the field suggests that M1, also known as pro-inflammatory macrophages are detrimental for the disease while M2, also known as anti-inflammatory and tissue repair macrophages might have a protective role, driving the joint to homeostasis.^26^ Moreover, it has been suggested that drugs that alter macrophage phenotype from M1 to M2 would be an effective treatment for OA.^27^ However, our findings suggest that not only pro-inflammatory but also tissue repair macrophages contribute to chondrocyte catabolism.

Here we report that chondrocyte hypertrophy is not necessarily promoted in cultured human chondrocytes by pro-inflammatory signaling cues, as was observed in mice. Attention should be paid to the difference between human and murine chondrocytes when looking for disease modifying drugs, as hypertrophic differentiation might be differently regulated. Our data suggest that targeting tissue repair macrophages might be used as a therapy to inhibit hypertrophy of human chondrocytes.

## Supplemental Material

sj-docx-1-car-10.1177_19476035211021907 – Supplemental material for Effect of Inflammatory Signaling on Human Articular Chondrocyte Hypertrophy: Potential Involvement of Tissue Repair Macrophages Click here for additional data file. Supplemental material, sj-docx-1-car-10.1177_19476035211021907 for Effect of Inflammatory Signaling on Human Articular Chondrocyte Hypertrophy: Potential Involvement of Tissue Repair Macrophages by Mauricio N. Ferrao Blanco, Yvonne M. Bastiaansen-Jenniskens, Mark G. Chambers, Andrew A. Pitsillides, Roberto Narcisi and Gerjo J.V.M. van Osch in CARTILAGE
