# Supplementary material for: Effect of Inflammatory Signaling on Human Articular Chondrocyte Hypertrophy: Potential Involvement of Tissue Repair Macrophages
Source: Cartilage. 2021 Jun 24;13(2 Suppl):168S–174S. doi: 10.1177/19476035211021907 (PMC8739598; doi:10.1177/19476035211021907)
Supplement: sj-docx-1-car-10.1177_19476035211021907 – Supplemental material for Effect of Inflammatory Signaling on Human Articular Chondrocyte Hypertrophy: Potential Involvement of Tissue Repair Macrophages [file sj-docx-1-car-10.1177_19476035211021907.docx]

**Supplementary figure**


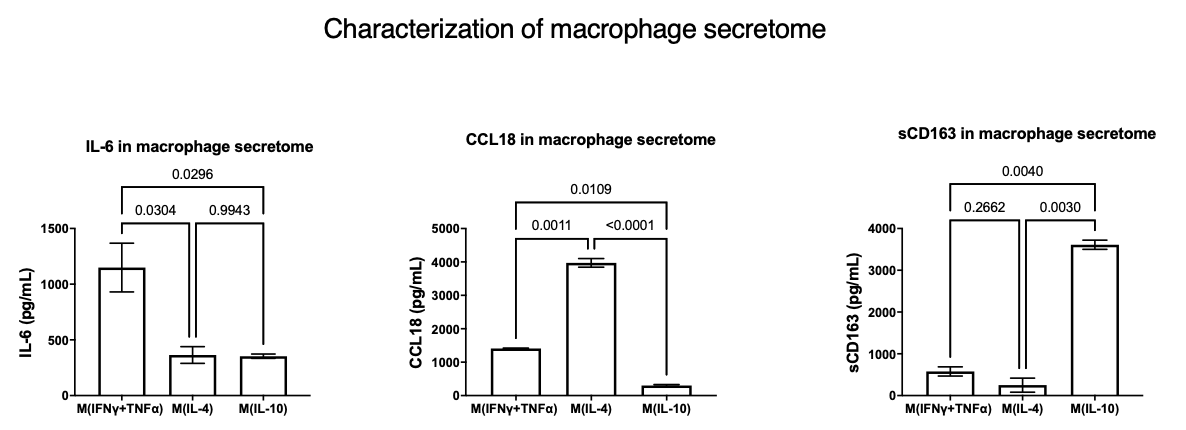


**Supplementary figure 1.** Characterization of macrophage secretome. Interleukin (IL)- 6, C-C Motif Chemokine Ligand (CCL) 18 and soluble (s) Cluster of differentiation (CD) 163 quantified by enzyme-linked immunosorbent assay (ELISA) in the medium conditioned by monocyte-derived macrophages. Data is shown as mean ± SD.

**Supplementary figure 2.** Nitrite concentration in the medium of OA human chondrocytes in 2D (A) and in alginate (B) stimulated with pro-inflammatory cytokines for one week (n=3 donors, 3 samples per donor). Data is shown as mean ± SD.
